# Literary Notes

**Published:** 1898-08

**Authors:** 


### LITERARY NOTES.

THE transactions of the Medical Society of the State of New York, for the session of 1898, is just issued from the press. The volume contains 500 pages, has a discussion on *x*-rays in medicine and surgery, one on diseases of the prostate, forty-four timely articles, minutes of the meeting, reports of committees, revised county society lists, besides other interesting contributions. The price to members of the society is $1.25, to others $1.50. Communications should be addressed to Dr. F. C. Curtis, secretary, 17 Washington avenue, Albany.

---

THE beautiful brochure, entitled "The Ship's Doctor," which is being issued to physicians by The Arlington Chemical Co., of Yonkers, N. Y., is of unique interest. Nor is this interest due solely to the novelty of the subject; for, independently of this, the booklet is notable as marking the highest point yet reached in certain features of artistic bookmaking. The deadly battle horrors of the surgeon's merciful vocation are full of dramatic opportunities for the artist, but only an artist of power can make such gruesome scenes impressive instead of merely horrible. Mr. W. Granville Smith is such an artist and he has made for "The Ship's Doctor" a series of battle pictures which touch the highest mark of the illustrator's art. A great naval battle is depicted with thrilling realism and the grim realities of war are uncovered by portrayals of the cock-pit during an action and of episodes of the surgeon's battle duties.

The beauty of this booklet, its professional interest and its timeliness, are certain to make a lively call for it, and physicians who have not received a copy should at once send for it, as the edition is limited and will be issued in the order as requests are received. The more important pictures are admirable subjects for framing and if there are received a number of requests sufficient to warrant the great expense, a series of plates in large size, with liberal margins suitable for framing, will be made and supplied free to physicians. Physicians who would like to have them for framing should make their requests to The Arlington Chemical Co., Yonkers, N. Y., makers of liquid peptonoids, without loss of time.

---

THE J. B. Lippincott Company, of Philadelphia, publish a useful blood chart, designed by Dr. J. C. Da Costa, Jr., M. D. In these days the examination of the blood becomes important in the diagnosis of many diseases, hence it is convenient to have such a chart as this at hand to record observations.

---

THE partnership hitherto existing between Presley Blakiston and Kenneth M. Blakiston, under the firm name of P. Blakiston, Son & Co., expired June 30, 1898, on account of the death of the senior member. The business of publishing, importing and dealing in medical and scientific books, as established in 1843, will be continued by Kenneth M. Blakiston, trading as P. Blakiston's Son & Co.

THE *Canadian Journal of Medicine and Surgery*, published in its July issue a special greeting to the International Association of Railway Surgeons, which included a three-color supplement and a poem of welcome, written by one of the editorial staff, Dr. E. H. Stafford. This enterprise no doubt will meet the approval of the patrons of this journal, including its exchanges.

---

Two new medical journals appeared during June, 1898, the *St. Louis Medical Gazette*, edited by Dr. Martin F. Engman and a staff of ten associates, and the *Medical and Surgical Monitor*, Indianapolis, edited by Dr. Samuel E. Earp.

